# P-1328. Stenotrophomonas maltophilia Infection Characteristics in Patients with Liver Disease

**DOI:** 10.1093/ofid/ofaf695.1516

**Published:** 2026-01-11

**Authors:** Kenneth Schlabach, Ashlan J Kunz Coyne

**Affiliations:** University of Kentucky College of Pharmacy, Lexington, KY; University of Kentucky College of Pharmacy, Lexington, KY

## Abstract

**Background:**

Bacterial infections are a leading complication in liver disease, often resulting in severe outcomes due to immune dysfunction and frequent healthcare exposure, often involving broad-spectrum antibiotics. *Stenotrophomonas maltophilia* is increasingly recognized in patients with cirrhosis but remains poorly characterized. This study aims to elucidate the clinical features, comorbidities, and prior antibiotic exposures associated with *S. maltophilia* infections in patients with liver disease to inform targeted management strategies.

**Table 1.**
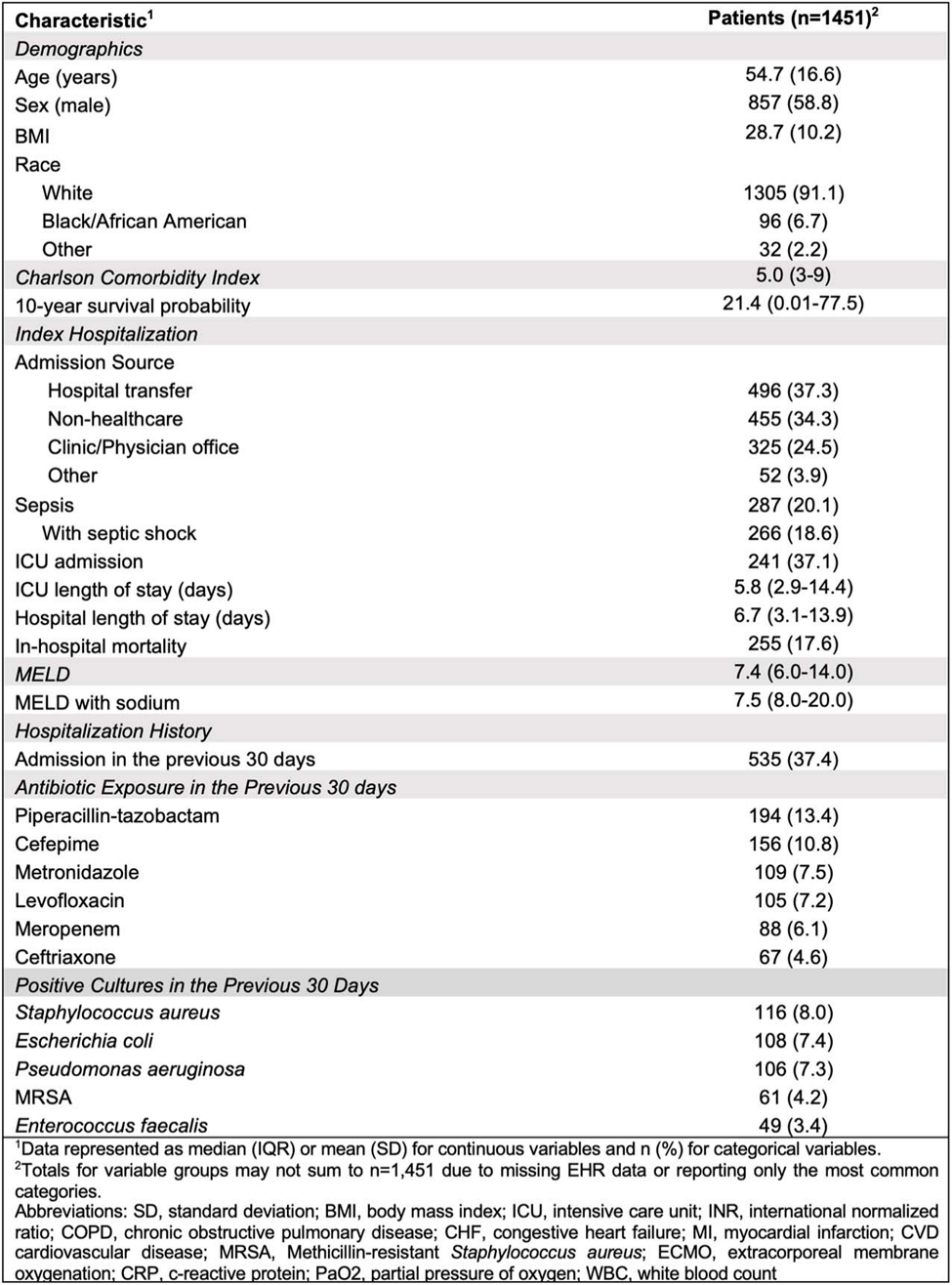
Demographics and Clinical Characteristics of Patients with Liver Disease and Stenotrophomonas maltophilia Infections

**Figure 1:**
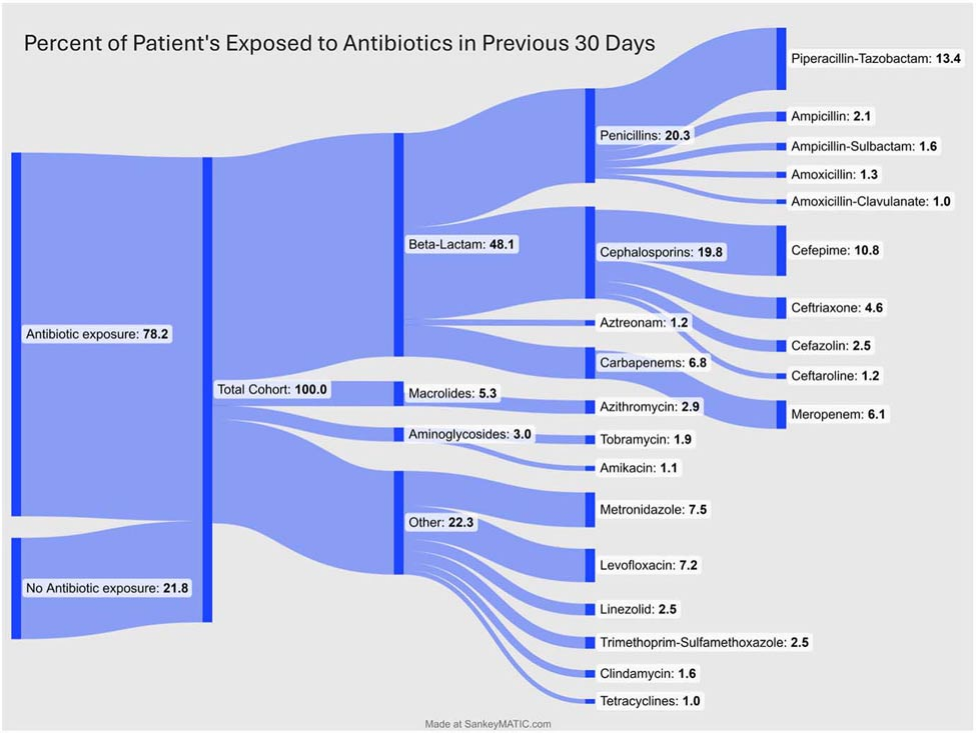
Sankey Diagram of Antibiotic Exposure 30 Days Preceding Cultures

**Methods:**

A retrospective cohort study at University of Kentucky HealthCare (2010–2023) analyzed unique adult patients with liver disease and culture-confirmed *S. maltophilia* infections from any clinical site. Data on clinical characteristics, comorbidities, prior 30-day antibiotic exposure, and outcomes were collected via standardized electronic health record extraction and chart review.

**Figure 2:**
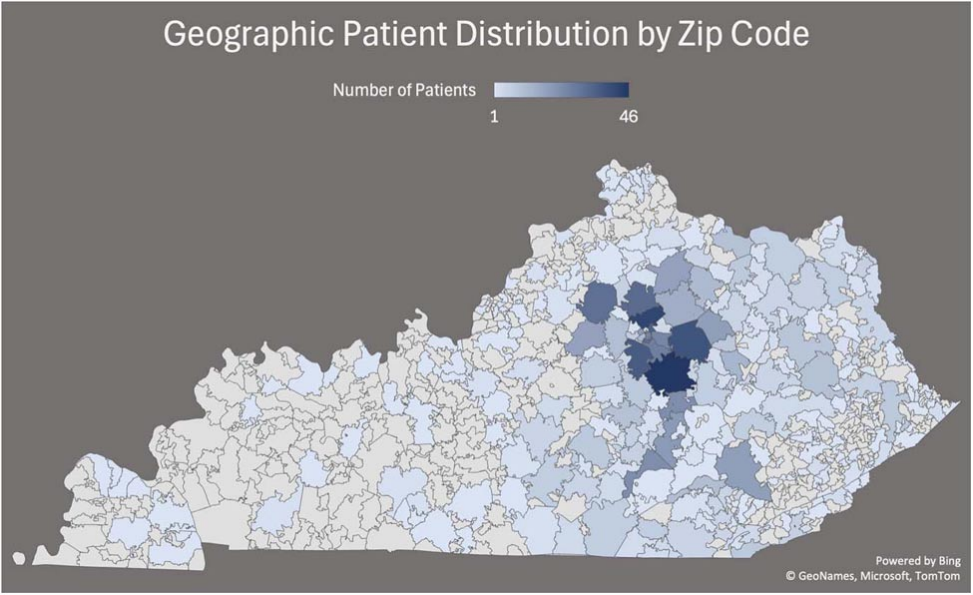
Geographic Patient Distribution by Zip Code

**Results:**

In-hospital mortality was 17.6% among 1,451 patients with liver disease and *S. maltophilia* infections (Table 1). The median MELD score was 7.4 (IQR 6.0–14.0), and Charlson Comorbidity Index was 5.0 (IQR 3–9), with a 10-year survival probability of 21.4%. Severe sepsis with shock occurred in 18.6%, and 37.1% required ICU care (median ICU LOS 5.8 days, IQR 2.9–14.4); hospital LOS was 6.7 days (IQR 3.1–13.9). Prior 30-day hospital admission occurred in 37.4%, and 78.2% had antibiotic exposure, most commonly piperacillin-tazobactam (13.4%) and cefepime (10.8%) (Figure 1). Prior cultures frequently grew *Staphylococcus aureus* (8.0%) and *Escherichia coli* (7.4%). Geographic distribution of admissions is shown in Figure 2.

**Conclusion:**

*S. maltophilia* infections in liver disease patients are linked to high prior antibiotic exposure, severe illness, and notable mortality. These findings underscore the need for early recognition, targeted prevention, and antimicrobial stewardship among patients with cirrhosis and advanced liver disease.

**Disclosures:**

All Authors: No reported disclosures

